# Management of Sternal Segment Dislocation in a Child with Closed Reduction

**DOI:** 10.1155/2012/676873

**Published:** 2012-03-14

**Authors:** Omer Soysal, Osman Cemil Akdemir, Sedat Ziyade, Murat Ugurlucan

**Affiliations:** ^1^Department of Thoracic Surgery, Medical Faculty, Bezmi Alem Foundation University, Vatan Caddesi, Capa, 34390 Fatih/Istanbul, Turkey; ^2^Cardiovascular Surgery Clinic, Anadolu Medical Center, Istanbul, Turkey

## Abstract

Trauma may lead to sternal fracture or dislocation. Dislocation of a sternal segment in the childhood period is very rare as for sternal fractures in children. There are only six case reports regarding the issue in the literature. Additionally, there is not an established consensus for the treatment of the pathology. In this paper we present traumatic dislocation of a sternal body segment in a 10-year-old child who was successfully managed conservatively by closed reduction together with the review of the literature. Surgical treatment is not necessary especially in acute cases. Pathology may be treated with closed reduction. Callus formation usually supports the dislocated part of the sternum in time.

## 1. Introduction

Dislocation of a sternal segment in the childhood period is very rare as for sternal fractures in children. There are only six case reports regarding the issue in the literature. Additionally, there is not an established consensus for the treatment of the pathology.

In this paper, we present our single case experience in which we applied conservative treatment which had been successful for the management of the pathology in the child.

## 2. Case Report

**Figure 1:**
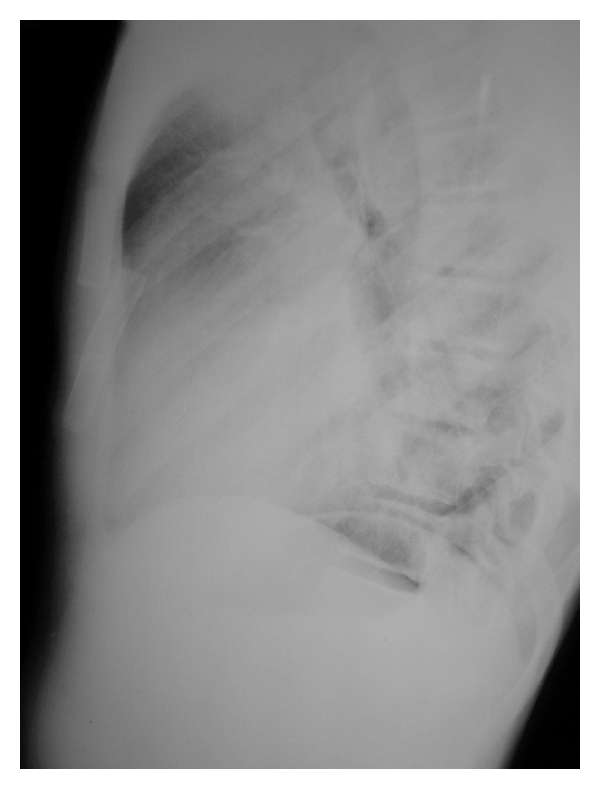
Lateral chest X-ray. Dislocated sternal segments.

A ten-year-old male patient was admitted to the emergency room due to fall on a blunt-ribbed body. He had chest pain. Physical examination showed a deformity at the sternum. There was tenderness and a click sound at palpation of the sternum. Posteroanterior chest X-ray was normal. At the lateral chest radiograph, sternal corpus was consisted of four segments. The second and third segments were dislocated together; the second one anteriorly and the third posteriorly (Figure 1). Electrocardiography revealed no pathology.

**Figure 2:**
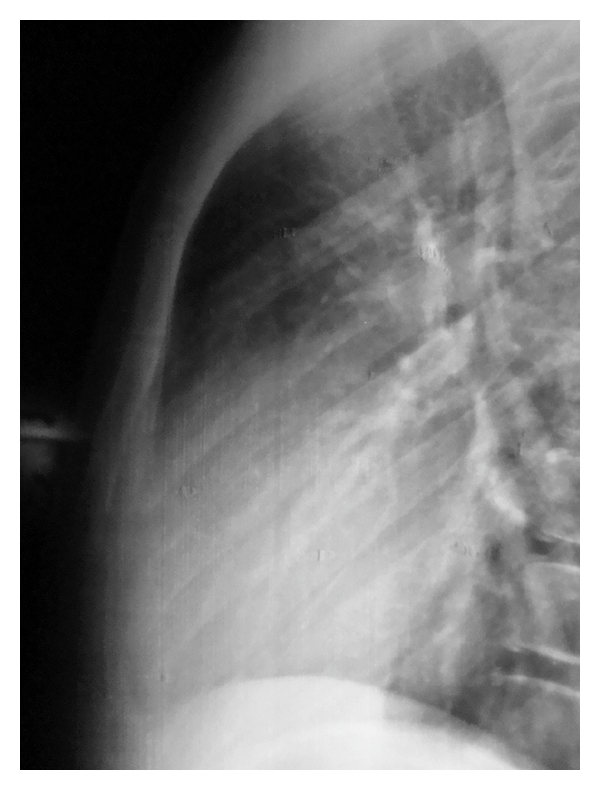
Lateral chest X-ray after reduction of the dislocated sternum.

For the treatment, the body of the child was supported from the ridge and the shoulders were stretched to the back. By means of this extension, reduction of the dislocated segments of the sternum was performed (Figure 2). After reduction, pain and the deformity over the sternum disappeared. The patient was followed for one day and discharged from the hospital. No pain, shortness of breath, or any other chest complaint developed, and the sternum appeared intact on the lateral chest radiograph.

**Figure 3:**
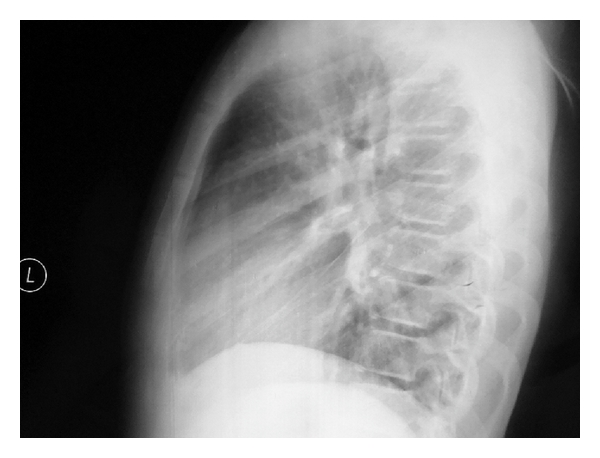
Lateral chest-X-ray after 6 months of reduction. There is a callus-like formation around the dislocated and deducted part of the sternum.

A lateral chest X-ray revealed no dislocation after 15 days. We observed a calcification at the corpus of the sternum between the first and the second segments on chest X-ray after three months, and the sternum was stable at palpation. After six months, lateral chest X-ray showed no dislocation, but a callus-like formation at the periarticular area of the segments one and two (Figure 3). The patient is followed symptom free, without any growth abnormalities for more than 9 months.

## 3. Discussion

Sternal fracture and sternal dislocation are different entities. However, clinical symptoms, signs, and treatment may be similar. Sternal dislocation may present as manubriosternal joint dislocation (MSJD) or sternal segment dislocation (SSD).

Recent reviews of isolated traumatic sternal fracture have shown that this injury constitutes an almost “benign entity” and the value of routine hospitalization and monitoring of these patients were questioned [1]. Several centers in the UK discharge selected patients with sternal fracture directly from the emergency department. They are admitted for a minimum 24 hours [1]. In our facility, all patients with sternal fracture or sternal dislocation are hospitalized in the thoracic surgery department minimally for 24 hours. Our patient had a dislocation of a segment of the sternal corpus. Dislocation was treated with a simple extension maneuver, and the child was followed up for cardiac injury, hemorrhage, pain, and redislocation for one day.

There have been few reports of fractures of the sternum in children. The sternum is anatomically protected from fracture by surrounding ligaments and cartilaginous attachments to the ribs. In children, these structures are even more elastic and the ribs are more flexible than in adults, making a child's sternum even less susceptible to fracture. Dislocation of a sternal segment in children also is a very rare event. The segments start to ossify early in childhood, but synostosis formation between the segments starts in the seventh year of life [2]. If a dislocated sternal segment is deducted without any surgery and could be stabilized for a while, a redislocation does not occur, as we know from the scenario of sternal fractures.

There are two types of MSJD. Type I is a backward dislocation of the body caused by a direct force acting on it, for example by direct compression injury to the anterior chest. Type II, which is the most common, follows hyperflexion with compression injury to the upper thorax. Indirect forces transmitted to the sternum through the clavicles, the chin, or the upper two ribs cause backward displacement of the manubrium. Tremendous force is required to fracture or dislocate the MSJ. The diagnosis of MSJ disruption is suspected clinically and detected by a lateral roentgenogram of the thorax [1].

Differential diagnosis from sternal fracture and the diagnosis of sternal dislocation are based on physical examination, deformity and pain with palpation of the sternum, and lateral chest X-ray. Ultrasonography and computed tomography may also give detailed information about the sternal pathology, the mediastinum and the lung. Ultrasonography may be better than lateral chest X-ray to diagnose sternal fractures and dislocations [1].

Five cases of sternal segment dislocation were reported in the literature in the pediatric age group. Due to the small number of cases, a standard protocol for treatment is not available. Woo [3] reported a successful treatment with reduction by manipulation in the sternum with hyperflexion. Watanabe and colleagues [2] reported a sternal segment dislocation in a 3-year-old child who was treated with a new implant by means of open reduction. Watanabe et al. [4] reported a 5-year-old child with sternal segment dislocation treated with closed reduction which redislocated after six days. That child was followed up for one year. The dislocated and rotated sternum remodeled and improved with a full recovery [4]. The course of the remodeled sternum of this patient and improvement with callus formation of our patient dictated that surgical fixation may not be necessary for sternal segment dislocation in children. Suzuki et al. [5] also reported that segment dislocation could be reduced immediately after injury.

In conclusion, sternal segment dislocation during the childhood period is extremely rare. Surgical treatment is not necessary especially in acute cases. Pathology may be treated with closed reduction. Callus formation usually supports the dislocated part of the sternum in time.
